# Cognitive-Enhancing Effect of Quercetin in a Rat Model of Parkinson's Disease Induced by 6-Hydroxydopamine

**DOI:** 10.1155/2012/823206

**Published:** 2011-07-18

**Authors:** Napatr Sriraksa, Jintanaporn Wattanathorn, Supaporn Muchimapura, Somsak Tiamkao, Kamoltip Brown, Kowit Chaisiwamongkol

**Affiliations:** ^1^Department of Physiology and Graduate School (Neuroscience Program), Faculty of Medicine, Khon Kaen University, Khon Kaen 40002, Thailand; ^2^Department of Physiology, Faculty of Medicine, Khon Kaen University, Khon Kaen 40002, Thailand; ^3^Department of Medicine, Faculty of Medicine, Khon Kaen University, Khon Kaen 40002, Thailand; ^4^Department of Anatomy, Faculty of Medicine, Khon Kaen University, Khon Kaen 40002, Thailand

## Abstract

Oxidative stress has been reported to induce cognitive impairment in Parkinson's disease. This paper aimed to determine the effect of quercetin, a substance possessing antioxidant activity, on the cognitive function in a rat model of Parkinson's disease. Male Wistar rats, weighing 200–250 g, were orally given quercetin at doses of 100, 200, 300 mg/kg BW once daily for a period of 14 days before and 14 days after the unilateral lesion of right substantia nigra induced by 6-hydroxydopamine (6-OHDA). Their spatial memory was assessed at 7 and 14 days of treatment and neuron density was determined, malondialdehyde (MDA) level, the activity of superoxide dismutase (SOD), catalase (CAT), and glutathione peroxidase (GPx) were evaluated at the end of the experiment. In addition, the activity of acetylcholinesterase (AChE) was also measured. It was found that all doses of quercetin enhanced spatial memory. Therefore, it is suggested that the cognitive-enhancing effect of quercetin occurs partly because of decreased oxidative damage resulting in increased neuron density.

## 1. Introduction

Parkinson's disease (PD) is the second most common neurodegenerative disease. To date, the precise mechanism responsible for pathogenesis of PD is not well understood. Accumulating lines of evidence have demonstrated that oxidative stress plays a crucial role on the pathogenesis of PD [1–5]. The motor impairment observed in patients seems to be the most notable point of concern when prescribing medicine or planning treatment. In recent years, it was indicated that nonmotor deficits became an important part of patient management [6]. It has been known that patients in the early stages of PD already have impairment of cognitive performance and this deficit in turn gives rise to a poor quality of life [7, 8] and economic burden [9]. Recently, it was found that 6-hydroxydopamine (6-OHDA) was recognized as a good model for early stages of PD, especially in terms of emotional and cognitive deficits [10]. Although the substantia nigra is the brain area that has been indicated to be the most vulnerable to neurodegeneration in PD and mediates the clinical manifestation (rigidity, resting tremor, slowness of voluntary movement, and postural instability), but other brain regions such as locus ceruleous, raphe neuclei, nucleus basalis of Meynert, and the brain area that responsible for cognition, hippocampus, were also affected. Previous MRI studies clearly revealed that decreased hippocampal volume accompanied the cognitive impairment in PD patients [11–13]. Based on the role of oxidative stress on the pathophysiology of PD, the neuroprotective and cognitive enhancing effects of substances possessing antioxidant activity have gained much attention. 

Quercetin (QC) is a polyphenolic compound found in common vegetables and fruits such as onions, broccoli, and apples. Previous studies have demonstrated that quercetin contains many good biological properties for human health including antioxidant [14], anti-inflammation [15], and anticancer [16] activities. Recently, it has been reported that quercetin can pass through the blood-brain barrier of in situ models [17]. In addition, quercetin exerts the protective effect in a stroke model induced by transient global ischemia [18]. Quercetin significantly protected the neuronal cells from the oxidative stress-induced neurodegeneration in Alzheimer's disease [19], decreased lipid peroxidation, improved the activity of catalase and superoxide dismutase [20] and also prevented glutathione depletion [21]. Previous study showed that the EGb761, a standardized extract from the herbal medicine* Ginkgo biloba,* contains a high amount of quercetin and exhibits the neuroprotective effect against oxidative damage induced by 6-OHDA [22]. Moreover, it was found that quercetin attenuated the neuronal death in the hippocampus resulting in improved learning and memory in arm maze test [23]. Therefore, these pieces of evidence point out the possibility that quercetin might exert an influence on the central nervous system. 

Based on the antioxidant and anti-inflammatory actions of QC, we hypothesized that QC might mitigate the neurotoxicity and cognitive impairment in an animal model of PD induced by 6-OHDA. To elucidate this issue, the current study aimed to determine the effects of QC on spatial memory and neuron density in the hippocampus of an animal model of PD induced by 6-OHDA. In addition, the possible underlying mechanism was also investigated. 

## 2. Materials and Methods

### 2.1. Animals

Adult male Wistar rats (10 weeks old) were obtained from the National Laboratory Animal Center, Salaya, Nakhon Pathom. The animals weighted between 200 to 250 grams at the beginning of experiment. They were housed 5 per cage and maintained in 12 : 12 light : dark cycle and given access to food and water *ad libitum*. The experiments were performed to minimize animal suffering in accordance with the internationally accepted principles for laboratory use and care of European Community (EEC directive of 1986; 86/609/EEC). The experimental protocols were approved by the Institutional Animal Care and Use Committee.

### 2.2. Drugs and Chemicals

Quercetin was purchased from Sigma-Aldridge Company. All chemical substances used in this study were analytical grade. Both Levodopa (L-dopa), a standard drug used for PD treatment, and vitamin C, an antioxidant possessing the neuroprotective and cognitive enhancing effects, were used as positive controls in this study. Normal saline solution (NSS) was used as vehicle throughout the study. All administered substances were freshly prepared.

### 2.3. Experimental Protocol

The animals were randomly divided into 7 groups (*n* = 8 animals/group) as described below. 

Group I: Control. Animals were not treated with any drugs or injections. 

Group II: Vehicle plus 6-OHDA injection. Animals were treated with NSS once daily via oral route for 14 days before and 14 days after the injection of 6-OHDA into the right substantia nigra. 

Groups III–V: Rats were treated with quercetin at various doses ranging from 100, 200, and 300 mg/kg BW once daily via oral route for 14 days before and 14 days after the injection of 6-OHDA into the right substantia nigra. 

Groups VI–VII: Rats were orally treated with either Levodopa (L-dopa; 5 mg/kg BW) or vitamin C (200 mg/kg) once daily for 14 days before and 14 days after the lesion of the right substantia nigra induced by 6-OHDA.

Each rat in all groups, except the control group, was orally given the same volume of substance suspension for 14 days. The rats were then subjected to 6-OHDA injection using the stereotaxic apparatus. The 6-OHDA infusion was performed using a Hamilton syringe and rats were allowed to recover after the lesion for 6 days. They were then tested for spatial memory using Morris water maze test at 7 and 14 days after 6-OHDA injection. At the end of experiment, rats were sacrificed and their brains were removed and the density of survival neurons, the activity of acetylcholinesterase (AChE), and scavenger enzymes including superoxide dismutase (SOD), catalase (CAT), and glutathione peroxidase (GPx) in the hippocampus were determined. Moreover, the malondialdehyde (MDA) level in the mentioned area was also determined.

### 2.4. The Administration of 6-OHDA

6-OHDA was prepared as described previously by Ferro and coworkers [24]. Rats were anesthetized with 50 mg/kg sodium thiopental (i.p.), each animal was mounted on a stereotaxic stand, the skin overlying the skull was cut to expose the skull, and the coordinates for the substantia nigra par compacta (SNpc) were accurately measured (anteroposterior −0.5 mm from bregma, mediolateral 2.1 mm from midline, and dorsoventral −7.7 mm from the skull). The 6 *μ*g of 6-OHDA, which dissolved in 2 *μ*L 0.2% ascorbic acid saline, were perfused into SNpc through a 30-gauge stainless needle. After the surgery, animals were allowed to recover from anesthesia and then placed in their cages.

### 2.5. Morris Water Maze Test

The water maze consisted of a metal pool (170 cm in diameter × 58 cm tall) filled with tap water (25°C, 40 cm deep). The pool was divided into 4 quadrants (NE, NW, SE, and SW) by two imaginary lines crossing the center of the pool. The water surface was covered with nontoxic milk. The removable platform was placed below the water level at the center of one quadrant. For each animal, the location of the invisible platform was placed at the center of one quadrant and remained there throughout training. The rats must memorize the platform location in relation to various environmental cues because the location of the escape platform in and outside the pool is not visible. Each rat was gently placed in the water facing the wall of the pool from one of the four starting points (N, E, S, or W) along the perimeter of the pool, and the animal was allowed to swim until it found and climbed onto the platform. During training session, the rat was gently placed on the platform when it could not reach the platform in 60 seconds. In either case, the subject was left on the platform for 15 seconds and then removed from the pool. The time for animals to climb on the hidden platform was recorded as escape latency or acquisition time. In order to determine the capability of the animals to retrieve and retain information, the platform was removed 24 hr later and the rat was released into the quadrant diagonally opposite to that which contained the platform. Time spent in the region that previously contained the platform was recorded as retention time. In each trial, the animal was quickly dried with towel before being returned to the cage [25].

### 2.6. Histological Procedure

Following anesthesia with sodium pentobarbital (50 mg/kg BW), the brain fixation was carried out by transcardial perfusion with a fixative solution containing 4% paraformaldehyde in 0.1 M phosphate buffer pH 7.3. After the perfusion, the brains were removed and stored overnight in a fixative solution that is used for perfusion. They were then infiltrated with 30% sucrose solution at, and kept at, 4°C. The specimens were frozen rapidly and the coronal sections at 30 *μ*M thick were prepared using cryostat. All sections were rinsed in the phosphate buffer and picked up on slides coated with 0.01% aqueous solution of a high molecular weight poly L-lysine.

### 2.7. Nissl Staining

The duplicate coronal sections of brains were stained with 0.75% cresyl violet, dehydrated through graded alcohols (70, 95, 100% 2x), placed in xylene, and mounted using DPX.

### 2.8. Determination of Acetylcholinesterase, Superoxide Dismutase, Glutathione Peroxidase and Catalase Activity, and the Malondialdehyde Level

The rats were divided into various groups as previously described in the experimental protocol. After the last dose of administration, all rats were sacrificed. The hippocampus of the lesion side was isolated and prepared as a homogenate to determine the MDA level and the activities of AChE, SOD, CAT, and GPx. MDA was estimated by determining the accumulation of thiobarbituric acid reactive substances (TBARS) [26] in the hippocampal homogenate whereas the activities of acetylcholinesterase [27], superoxide dismutase [28], glutathione peroxides [29], and catalase [30] were determined using the colorimetric method.

### 2.9. Statistical Analysis

Data are expressed as means ± S.E.M. and were analyzed statistically by one-way ANOVA, followed by post hoc (LSD) test. The results were considered statistically significant at *P* value < 0.05.

## 3. Results

### 3.1. Morris Water Maze Test

**Figure 1:**
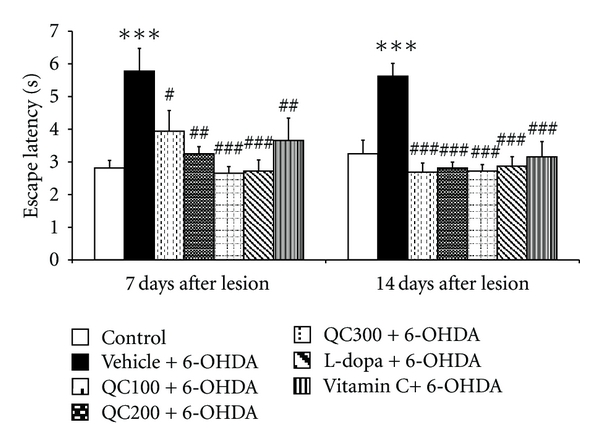
Effect of quercetin on escape latency in the Morris water maze test in an animal model of Parkinson's disease induced by 6-OHDA. Data were expressed as mean ± S.E.M. for 8 rats in each group. ***P < 0.001 compared to control; ^#^ *P* < 0.05; ^##^ *P* < 0.01; ^###^ *P* < 0.001 compared to the vehicle + 6-OHDA-treated group.

**Figure 2:**
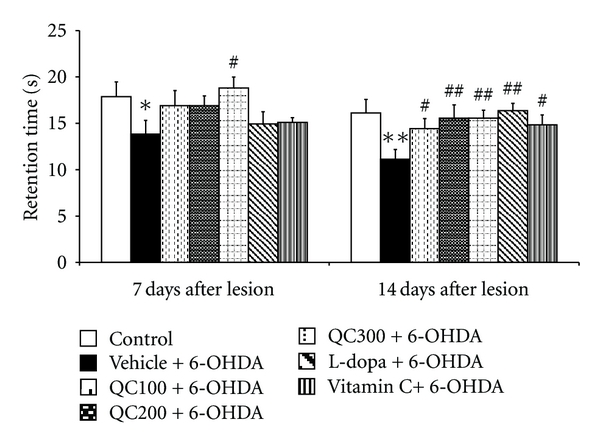
Effect of quercetin on retention time in the Morris water maze test in an animal model of Parkinson's disease induced by 6-OHDA. Data were expressed as mean ± S.E.M. for 8 rats in each group. *P < 0.05; **P < 0.01 compared to control; ^#^ *P* < 0.05; ^##^ *P* < 0.01 compared to the vehicle + 6-OHDA-treated group.

Cognitive impairment is commonly observed in Parkinson patients. Therefore, we examined the effect of quercetin on spatial memory by using the Morris water maze test and recorded escape latency and retention time as the indices. The results were shown in Figures 1 and 2.  Figure 1 demonstrates that 6-OHDA injection significantly increased escape latency (*P* < 0.001 compared to control). Both L-dopa and vitamin C could decrease escape latency throughout the observation period (day 7:* P* < 0.001 and 0.01, respectively, compared to vehicle+6-OHDA; day 14: *P* < 0.001 all, compared to vehicle+6-OHDA). At the 7th day after lesion, all doses of quercetin (100, 200, and 300 mg/kg BW) used in this study significantly decreased the escape latency (*P* < 0.05, 0.01, and 0.001, resp., compared to vehicle+6-OHDA). The beneficial effect of quercetin to improve learning and memory was also observed throughout the 14-day period after lesion (*P* < 0.001 all, compared to vehicle+6-OHDA). 

Figure 2 shows that 6-OHDA injection significantly decreased retention time (*P* < 0.05 and 0.01 at day 7 and day 14, resp., compared to control). However, only the high dose of quercetin significantly increased retention time at 7 days after lesion (*P* < 0.05 compared to vehicle+6-OHDA). When the treatment was prolonged further to 14 days after lesion, all doses of quercetin-treated groups could enhance retention time (*P* < 0.05, 0.01, 0.01 resp., compared to vehicle+6-OHDA) as similar as the positive modulation effect of L-dopa and vitamin C (*P* < 0.01 and 0.05 resp., compared to vehicle+6-OHDA).

### 3.2. Density of Survival Neuron in the Hippocampus

**Figure 3:**
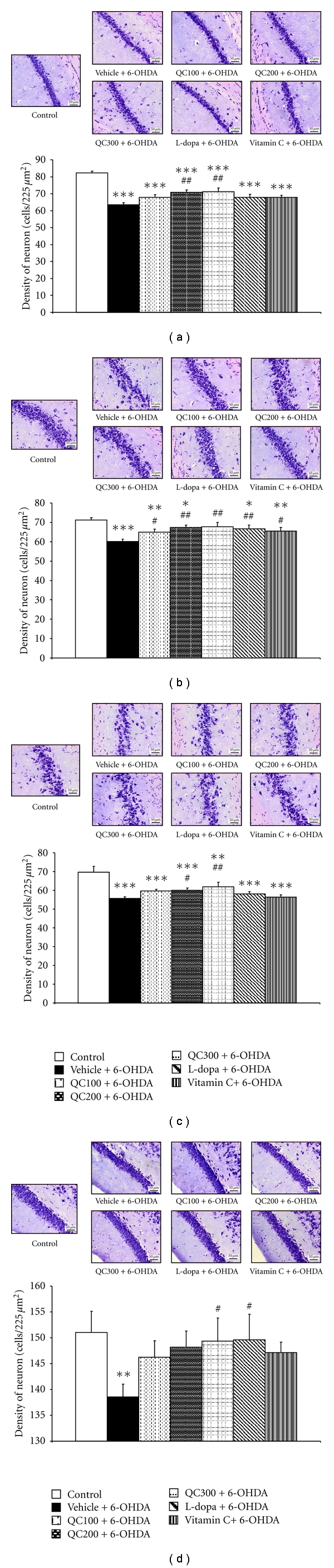
Effect of quercetin on neuron density in various subregions of the hippocampus (a = CA1; b = CA2; c = CA3; d = Dentate gyrus) in an animal model of Parkinson's disease induced by 6-OHDA. Scale bars 50 *μ*m, the magnification was 40X. Data were expressed as mean ± S.E.M. for 8 rats in each group. *P < 0.05; **P < 0.01; ***P < 0.001 compared to control; ^#^ *P* < 0.05; ^##^ *P* < 0.01 compared to the vehicle + 6-OHDA-treated group.

Previous information reported that the hippocampus plays a crucial role in learning and memory. Therefore, we also determined the effect of quercetin on the neuron density in the various subregions (CA1, CA2, CA3, and dentate gyrus) of the hippocampus. The results are shown in Figure 3. 6-OHDA injection significantly produced the neurodegeneration in all subregions of the hippocampus (*P* < 0.001, 0.001, 0.001, and 0.01 for CA1, CA2, CA3, and the dentate gyrus resp., compared to control). L-dopa-treated group significantly increased the neuron density in both CA2 and the dentate gyrus (*P* < 0.01 and 0.05 resp., compared to vehicle+6-OHDA) while vitamin C-treated group showed a significant increase of neuron density only in CA2 (*P* < 0.05 compared to vehicle+6-OHDA). Quercetin at a dose of 300 mg/kg BW significantly increased neuron density in all subregions of the hippocampus (*P* < 0.01, 0.01, 0.01, and 0.05 resp., compared to vehicle+6-OHDA). The medium dose of quercetin (200 mg/kg BW) significantly enhanced neuron density in CA1, CA2, and CA3 (*P* < 0.01, 0.01 and 0.05 resp., compared to vehicle+6-OHDA), while the low dose or quercetin at dose of 100 mg/kg BW could significant increase the neuron density in only CA2 (*P* < 0.05 compared to vehicle+6-OHDA).

### 3.3. Acetylcholinesterase Activity

**Figure 4:**
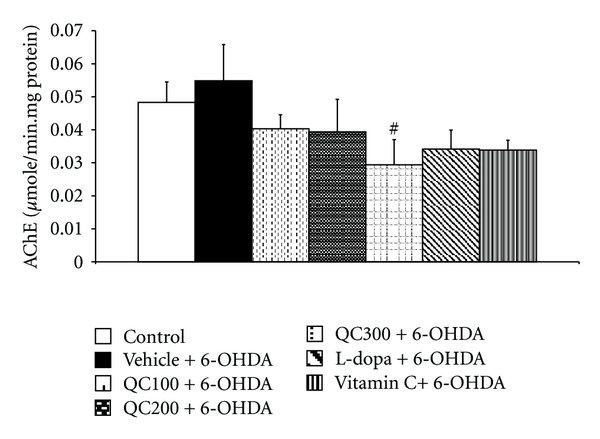
Effect of quercetin on the activity of acetylcholinesterase (AChE) in the hippocampus in an animal model of Parkinson's disease induced by 6-OHDA. Data were expressed as mean ± S.E.M. for 8 rats in each group. ^#^ *P* < 0.05 compared to the vehicle + 6-OHDA-treated group.

Based on the knowledge that cholinergic system plays the crucial role in spatial memory, the effect of quercetin on the activity of AChE, an indirect indicator of cholinergic function was evaluated. It was found that the high dose of quercetin significantly decreased the AChE activity in the hippocampal homogenate (*P* < 0.05 compared to vehicle+6-OHDA; Figure 4). However, the low and medium doses (100 and 200 mg/kg BW resp.) of quercetin and both positive control groups (L-dopa and vitamin C treatments) failed to produce significant alterations on this enzyme activity.

### 3.4. Determination of Malondialdehyde Level and Scavenging Enzymes Activities

**Figure 5:**
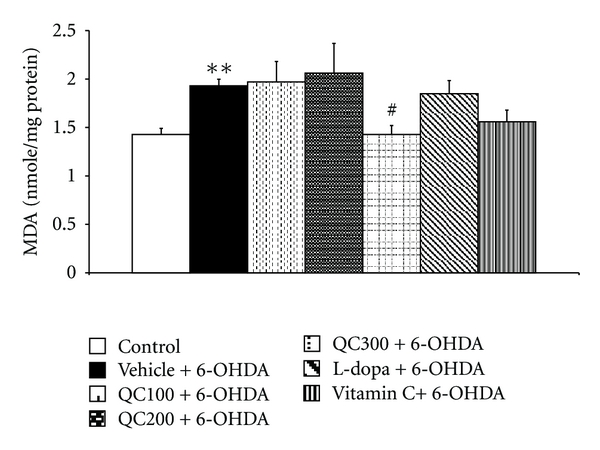
Effect of quercetin on the level of malondialdehyde (MDA) in the hippocampus in an animal model of Parkinson's disease induced by 6-OHDA. Data were expressed as mean ± S.E.M. for 8 rats in each group.**P < 0.01 compared to control; ^#^ *P* < 0.05 compared to the vehicle + 6-OHDA-treated group.

**Figure 6:**
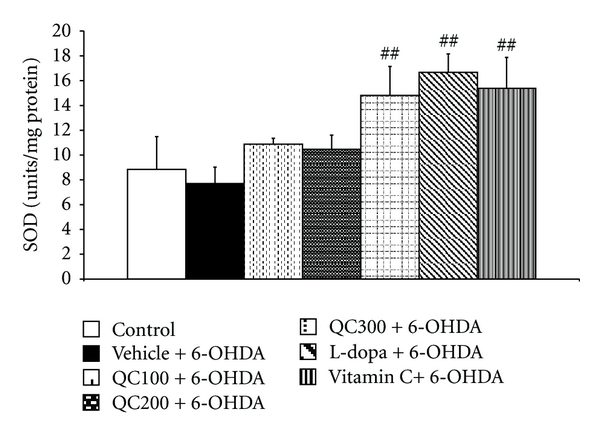
Effect of quercetin on the activity of superoxide dismutase (SOD) in the hippocampus in an animal model of Parkinson's disease induced by 6-OHDA. Data were expressed as mean ± S.E.M. for 8 rats in each group. ^##^ *P* < 0.01 compared to the vehicle + 6-OHDA-treated group.

**Figure 7:**
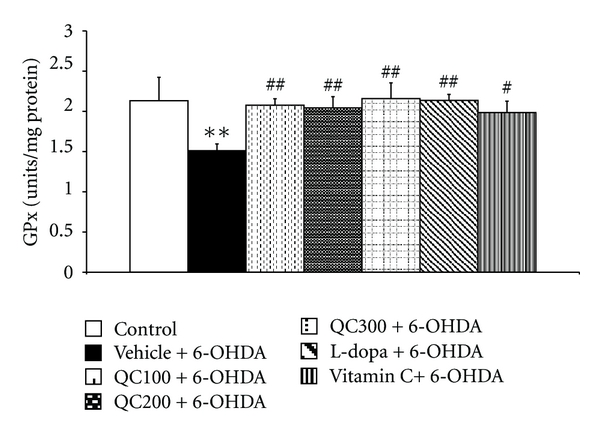
Effect of quercetin on the activity of glutathione peroxidase (GPx) in the hippocampus in an animal model of Parkinson's disease induced by 6-OHDA. Data were expressed as mean ± S.E.M. for 8 rats in each group. **P < 0.01 compared to control; ^#^ *P* < 0.05; ^##^ *P* < 0.01; compared to the vehicle + 6-OHDA-treated group.

**Figure 8:**
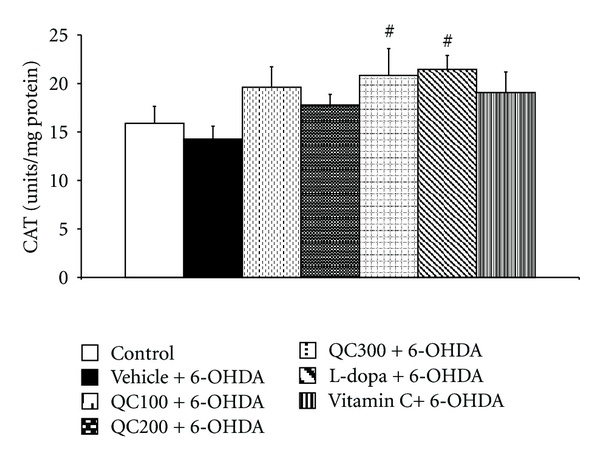
Effect of quercetin on the activity of catalase (CAT) in the hippocampus in an animal model of Parkinson's disease induced by 6-OHDA. Data were expressed as mean ± S.E.M. for 8 rats in each group. ^#^ *P* < 0.05 compared to the vehicle + 6-OHDA-treated group.

The right hippocampus was isolated in order to determine MDA levels and scavenging enzyme activities. The results are shown in Figures 5, 6, 7, and 8. It was found that 6-OHDA injection significantly increased MDA level (*P* < 0.01 compared to control) but decreased the activity of GPx (*P* < 0.01 compared to control). Both positive-control-treated groups failed to show a significant reduction of MDA levels in the hippocampus while the high dose of quercetin used in this study significantly decreased MDA levels in the mentioned area (*P* < 0.05 compared to vehicle+6-OHDA). In addition, both L-dopa and vitamin C also increased the activities of SOD (*P *< 0.01 all, compared to vehicle+6-OHDA), GPx (*P* < 0.01 and 0.05 resp., compared to vehicle+6-OHDA). However, only rats subjected to L-dopa showed significantly increased CAT activity (*P* < 0.05 compared to vehicle+6-OHDA). Interestingly, it was found that the rats subjected to the high dose of quercetin had significantly enhanced SOD, CAT, and GPx activity in the hippocampus (*P* < 0.01, 0.01, and 0.05 resp., compared to vehicle+6-OHDA). The medium and low doses of quercetin-treated groups also had significantly increased GPx activity in the aforementioned area (*P* < 0.05 all, compared to vehicle+6-OHDA).

## 4. Discussion

The results of this study clearly show the cognitive enhancing effect of quercetin in this PD model. It was found that treatment with quercetin for two weeks before and after a 6-OHDA injection can improve performance in the Morris water maze test of the rats. 

**Figure 9:**
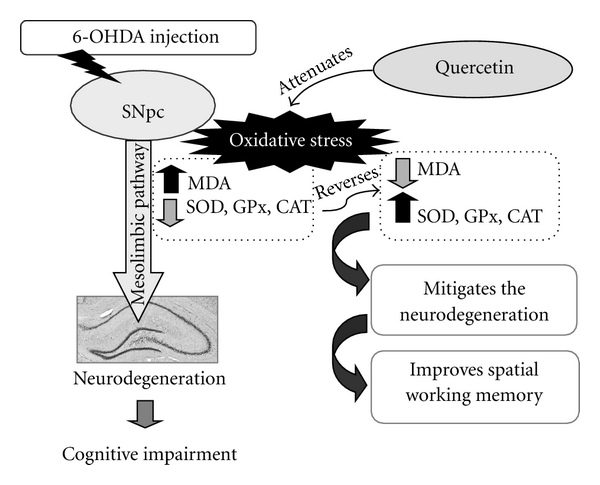
Hypothetical diagram of the possible underlying mechanism for the cognitive enhancing effect of quercetin on an animal model of Parkinson's disease induced by 6-OHDA.

6-OHDA is one of the most common neurotoxins used in experiments in order to mimic Parkinsonism in rodents. The possible underlying mechanism of neurotoxicity induced by 6-OHDA has been reported to be related to the oxidative stress caused by the production of hydroxyl radicals during autoxidation [31–33] and the inhibition of complex I [34] resulting in excessive oxidative stress and leading to neuronal death. Previous studies demonstrated that 6-OHDA could produce the cognitive deficit in animals [35–39], and oxidative stress has been shown to play an important role in memory impairment [40]. Thus, 6-OHDA is suitable to be used to mimic Parkinsonian and also cognitive impairment in rats (see Figure 9).

It is well known that the substantia nigra is the area of the brain that is most affected in PD but other brain areas are also affected. The hippocampus is the brain area that plays an important role in spatial memory [41, 42], thus we determined the cognitive performance of the rats using the Morris water maze test which is the best tool to determine spatial learning and memory in rodents [43]. 

We found that quercetin significantly improved the cognitive impairment induced by 6-OHDA injection, indicated by the decrease of time spent to find out platform (escape latency) and the increase of retention time. 

It has been reported that there was a tight correlation between cognitive impairment in PD and cholinergic deficit [44–46]. Therefore, we also determined the activity of AChE, an indirect indicator to evaluate the cholinergic system. The results of our study demonstrated that quercetin in a high dose significantly decreased AChE activity in the hippocampal homogenate, which indicates that there is the increase of available acetylcholine at the synaptic terminal resulting in the improvement of cognitive performance by the animals. 

It was found that in Parkinson patients' brain, there is an increase of lipid peroxidation [47, 48] and a decrease in the level of free radical scavenging enzymes [49]. The oxidative stress-induced neuronal death is the manner of 6-OHDA model. Therefore, we evaluated the density of survival neurons in the hippocampus. The results demonstrated that quercetin attenuated the neurodegeneration in all subregions of the hippocampus. In addition, we also determined the level of MDA and the activities of scavenging enzymes. The results of the present study demonstrate that 6-OHDA injection causes a significant increase of MDA levels but causes a significant decrease of scavenging enzymes indicating that free radicals are effectively involved in the development of cognitive impairment in PD. 

It is not clear how 6-OHDA produces oxidative stress in the hippocampus. Based on the knowledge that there are four main pathways of dopaminergic system, one of them is mesolimbic pathway which connects between the ventral tegmental area and the limbic system and includes the hippocampus. Moreover, it has been shown that the dentate gyrus of the hippocampus received the dopaminergic projection form the ventral tegmental area (A10) and the substantia nigra (A9) [50, 51]. 

In the present study, we injected 6-OHDA into the substantia nigra and because the nearby structure ventral tegmental area contains dopaminergic neuron thus these neurons might uptake the toxin into their cells too. Therefore, the dopaminergic connection to the hippocampus might be affected leading to the oxidative stress and cognitive impairment observed in the animals. Even though we observed the neuroprotective effect of quercetin in this study, we could not ignore recent information showing the pro-neurotoxic effect of quercetin if there is coexposure with methylmercury [52]. 

However, the results obtained from this study failed to show a dose-dependent manner. The possible explanation could be related to the rapid metabolism of quercetin [53]. Therefore, we observed the positive results only in the high-dose-treated group but not in the lower two doses because the active metabolites of quercetin might not be able to reach to therapeutic level and produce the significant effect on the parameters measured by this experiment. 

Previous studies strongly emphasized that quercetin is considered as a surpass free-radical scavenging antioxidant [54–56] owing to a high number of hydroxyl groups and an ability to donate electrons or hydrogens, and scavenge hydroxyl groups, hydrogen peroxide, and superoxide anions [57]. It was found that quercetin reverses the cognitive deficit induced by chronic reserpine administration [58]. The mechanisms behind this action could be a direct or indirect effect of quercetin to scavenge free radicals and oxidize metabolites or from iron or copper chelating and inhibit lipid peroxidation properties [59, 60]. Quercetin was reported to promote glutathione peroxidase [61], reverse the decrease of antioxidant defense of glutathione peroxidase, catalase, and superoxide dismutase induced by UVA light [62], and inhibit the hydrogen peroxide-induced oxidative damage [63, 64]. 

Our results suggest that the cognitive-enhancing effect of quercetin might be due to its antioxidant effect by promoting the activities of scavenging enzymes to protect the neurons from oxidative damage contributing to the survival of neurons in the hippocampus. However, we also observed a slight decrease in antioxidant enzymes in normal control rats. This is possibly due to the prooxidant effect of quercetin in normal cells [65].

Notably, only quercetin in high doses significantly decreased AChE activity resulting in the increase of available acetylcholine, an important neurotransmitter, which plays the crucial role in learning and memory process. However, the results in this study have also shown the beneficial effect on memory in the lower two doses of quercetin (100 and 200 mg/kg BW) but they failed to produce the significant suppression on AChE. We suggest that the improvement of cognition is likely to be related with an increase of neuron density.

## 5. Conclusion

It was concluded that quercetin exerts the cognitive enhancing effect in this PD model via its antioxidant effect resulting in the promotion of neuron survival. Consequently, the use of quercetin as an adjuvant therapeutic agent for the treatment of cognitive impairment in PD should be considered. However, further investigations are still required.
